# Clearing the HIV reservoir by transplanting CCR5 deficient stem cells

**DOI:** 10.1186/1742-4690-8-S2-O32

**Published:** 2011-10-03

**Authors:** Gero Hütter

**Affiliations:** 1 Institute of Transfusion Medicine and Immunology, Heidelberg University, Germany

## 

Today, 30 years after the appearance of the HIV pandemic, treatment strategies have considerably improved but there is still no cure for this disease available. Recently, we have described a successful haematopoietic stem cells transplantation in an HIV-1 infected patient applying donor cells with a natural resistance against this infection. These haematopoietic stem cells engrafted, proliferated and differentiated into mature myeloid and lymphoid cells and at present, the patient is more than 4 years after transplantation without the need of any antiretroviral treatment. Analyzing peripheral blood cells and different tissue samples, no viral load or proviral DNA could be detected. This case raises the hope for targeted treatment strategies against HIV and represents the first successful personalized allogeneic treatment with stem cells carrying a beneficial gene. However, this case has candled a discussion on the question whether this patient has achieved complete eradication of HIV. Here, we give an update on open questions, unsolved aspects, and consequences concerning this unique case report.

